# Cytoskeleton as an Emerging Target of Anthrax Toxins

**DOI:** 10.3390/toxins4020083

**Published:** 2012-02-06

**Authors:** Yannick Trescos, Jean-Nicolas Tournier

**Affiliations:** 1 Unité Interactions Hôte-Agents pathogènes, Institut de Recherche Biomédicale des Armées, Centre de Recherche du Service de Santé des Armées, BP 87, 24 avenue des Maquis du Grésivaudan 38702 La Tronche Cedex, France; Email: yannick.trescos@yahoo.fr; 2 Ecole du Val-de-Grâce, 1 place Alphonse Lavéran, 75005 Paris, France

**Keywords:** anthrax toxins, cytoskeleton, actin, phagocytosis, vascular integrity

## Abstract

*Bacillus anthracis*, the agent of anthrax, has gained virulence through its exotoxins produced by vegetative bacilli and is composed of three components forming lethal toxin (LT) and edema toxin (ET). So far, little is known about the effects of these toxins on the eukaryotic cytoskeleton. Here, we provide an overview on the general effects of toxin upon the cytoskeleton architecture. Thus, we shall discuss how anthrax toxins interact with their receptors and may disrupt the interface between extracellular matrix and the cytoskeleton. We then analyze what toxin molecular effects on cytoskeleton have been described, before discussing how the cytoskeleton may help the pathogen to corrupt general cell processes such as phagocytosis or vascular integrity.

## 1. Introduction

*Bacillus anthracis*, the agent of anthrax, is a common veterinary disease and major agent of biological warfare important for biodefense [1]. The attacks in 2001, consisting of letters contaminated with anthrax spores in the United States, have confirmed its potential use as a weapon of bioterrorism and justify the growing need to understand the pathophysiology of this dreadful pathogen [2]. *B. anthracis* is a gram-positive, aerobic bacterium that can form very resistant spores in poor environments. Its virulence is linked to two main factors: the capsule formed by poly γ-D-glutamic acid, whose operon is encoded by the plasmid pXO2, and the two toxins (edema toxin (ET) and lethal toxin (LT)) encoded on the other virulence plasmid named pXO1 [3,4]. By pulmonary route of infection, *B. anthracis* induces a toxemia associated with sepsis and respiratory distress within a few days, rapidly progressing to death without treatment [5,6]. Anthrax toxins play a central role in pathogenesis and deregulation of the immune system (review in [7,8]). At the cellular level, many toxin effects have been described (review in [4,9]). Some studies have suggested that the toxins also target the cytoskeleton [10,11,12,13,14,15]. We attempt here to review how the anthrax toxins may interact with the cytoskeleton, a crucial component of eukaryote cells crucially involved in homeostasis.

## 2. General Overview on *Bacillus anthracis* Toxins and the Cytoskeleton

### 2.1. *Bacillus anthracis* Toxins

**Figure 1:**
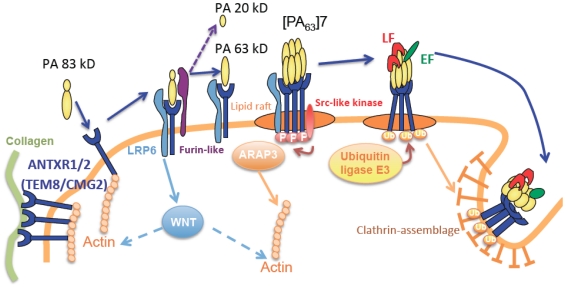
Interactions between anthrax toxin receptors and cytoskeleton. This figure highlights the links between cytoskeleton, anthrax toxin receptor (ANTXR) and the extracellular matrix. The physiological roles of ANTXR are not well known so far, unless they interact with extracellular matrix fibers. Their intracellular tail interacts with actin, and there is an inverse correlation between the binding of TEM8 to actin and the amount of protective antigen (PA) bound to receptors. After PA-ANTXR interaction, at least several signals can be sent to trigger PA endocytosis: (**i**) PA-ANTXR interactions trigger *src*-like kinase which in turn phosphorylates CMG-2 and TEM-8 intracellular tail favoring the toxin intracellular entry; (**ii**) a second target for anthrax receptor signaling may be ARAP3 (Arf GAP and Rho GAP with ankyrin repeat and pleckstrin homology domains); (**iii**) LRP6 coreceptor control Wnt signaling.

The toxins are A/B type, formed by the association of three components. Components A bear the enzymatic activity. Edema factor (EF, 89 kDa) for ET is a calmodulin-dependent adenylate cyclase, that increases intracellular cAMP concentrations [4,16], while lethal factor (LF, 90 kDa) for LT is a zinc-dependent metalloprotease cleaving specifically the *N*-terminus of most mitogen-activated protein kinase kinases (MAPKK or MEK) [4,17]. This MEK cleavage disrupts signaling cascades essential in cell proliferation, cell cycle regulation and immune function, such as ERK 1/2, JNK/SAPK and p38, signaling pathways (review in [4,7]). The B component, involved in binding cell receptor, is common for both toxins: it is named protective antigen (PA, 83 kDa), after its immunogenic properties (review in [9,18]). The mechanisms of cell penetration by toxins have been thoroughly deciphered over the past twenty years and can be separated into three major steps: receptor binding, internalization, membrane translocation (reviewed in [9,18]). PA_83 _binding to specific cell receptors (ANTXR1 or TEM-8 for Tumor Endothelial Marker-8 and ANTXR2 or CMG-2 for Capillary Morphogenesis Protein-2) [19] allows its cleavage by a furin-like protein into two subunits of 63 and 20 kDa, the latter being released (Figure 1). A third co-receptor named LDL receptor protein (LRP)-6 has been proposed [20], while its effective role has been discussed and may be cell-type specific [21,22]. Interestingly, a group has demonstrated that anthrax toxin receptors interact with LRP6 to control Wnt signaling [23]. 

PA_63_ subunits associate in heptamers to form a pre-pore, redistributed to the cell surface in lipid rafts. This allows the binding of EF or LF components in a stoichiometric ratio of 3 LF/EF to 7 PA. The toxin-receptor complex is then internalized by clathrin-dependent endocytosis [9,24,25]. A pH decrease within early endosomes results in the translocation of these factors in multivesicular bodies (MVB), finally merging with the intracellular membrane of late endosomes. This last step facilitates the translocation of LF into the cytoplasm, while EF stays associated to the membrane of the late endosome [26]. Its perinuclear localization generates intracellular cAMP gradients from the cell nucleus to the periphery. Interestingly, mouse mutants for each toxin receptor have been produced, showing that CMG-2 plays a unique role as a major receptor of anthrax toxin *in vivo* [27]. 

### 2.2. The Cytoskeleton

The cytoskeleton is a cellular scaffolding system whose functions include broad fundamental cell processes such as morphology and plasticity maintenance, movement, signal transduction, membrane and organelle trafficking. The cytoskeleton consists of three filament systems integrated into a complex network regulated by associated proteins: (i) actin microfilaments; (ii) microtubules; and (iii) intermediate filaments. As anthrax toxin effects have been described only on actin filaments and microtubules, we will restrict our description on these two systems.

Microfilaments are formed by a globular protein called actin, which exists in unpolymerized (G-actin) and polymerized (F-actin) forms. F-actin is composed of two parallels strands of actin monomers. The dynamic and spatial organization of the actin cytoskeleton is regulated at multiple levels by a variety of proteins that control numerous processes such as nucleation, polymerization, stabilization, branching and cross-linking of actin filaments [28,29,30]. Moreover, these regulatory and structural proteins enable cells to form and remodel functional structures like stress fibers, lamellipodia, filopodia, phagosomes and endocytic vesicles [30,31,32]. These macromolecular scaffolding structures are regulated by a complex interplay of Rho GTPases, kinases and phosphatases, which are again affected by bacterial pathogens or toxins [33,34]. The Rho GTPases are master regulators of cytoskeletal dynamics and cell shape [35], immune responses [36,37] and phagocytosis [38]. The three best-known members of the Rho GTPases family include RhoA, Cdc42 (Cell division Cycle 42) and Rac1, all of which can act as actin regulator switches by cycling between an inactive GDP- and active GTP-bound states [28,34]. In their active GTP-bound state, Rho GTPases interact with downstream effectors involved in the dynamic rearrangement of actin and microtubule filaments. Activation of RhoA induces the formation of stress fibers that are contractile bundles of polymerized actin containing myosin motor proteins. Rac1 regulates the formation of membrane ruffles or lamellipodia and Cdc42 controls the formation of filopodia or finger-like cell protrusions at the cell periphery [31,34,35].

Microtubules form well-organized hollow tubes composed of covalent association of alpha/beta tubulin heterodimers. These protofilaments radiate from the microtubule-organizing center (MTOC) located at the centrosome in the cytoplasm, in order to allow directional flow of proteins and organelles to a specific location [39,40]. The complex organization of microtubules is responsible for cell polarity, leading to MTOC’s movement toward the site of phagocytosis [41]. The Rho GTPases and many microtubule-associated proteins (MAPs), motor proteins like dyneins, and kinesins also interact with microtubules [37,40].

As the cytoskeleton intervenes in many dynamic cell processes, it has to be controlled by many regulatory proteins and is the target of multiple signaling pathways. As a result, the cytoskeleton network is a prime target for pathogens and their virulence factors. 

## 3. Association between Anthrax Receptor, Extra-Cellular Matrix and the Cytoskeleton

It is interesting to note that the precise physiologic role of TEM-8 and CMG-2 is currently unknown, although mutations of TEM-8 and CMG-2 in humans strongly suggest their role is very different from toxin endocytosis (review in [42]). Mutation in the *cmg-2* gene is linked to juvenile hyaline fibromatosis (JHF) and infantile systemic hyalinosis (ISH) [43,44]. Clinically, the patients suffer from generalized fibromatosis resulting by deposition of hyaline in the dermis. Mutation in the extracellular domain of *tem-8* has been described in a patient with infantile hemangioma, characterized by a rapidly growing area of angiogenesis [45]. Intriguingly, TEM-8 mutation disrupted the expression of VEGFR1 and VEGFR2 signaling. Taken together these data strongly suggests that TEM-8 and CMG-2 have very specialized functions hijacked by anthrax toxins. Along those lines the anthrax toxin triggers *src*-like kinase which in turn phosphorylates CMG-2 and TEM-8 intracellular domain favoring toxin entry [46]. A second target for anthrax receptor signaling may be ARAP3 (Arf GAP and Rho GAP with ankyrin repeat and pleckstrin homology domains) [47]. 

Extracellular domain of CMG-2 is known to interact with collagen IV and laminin [48]. Extracellular domain of TEM-8 interacts with collagen I and VI, and gelatin [15,49,50]. The cytosolic tail of TEM8 directly docks the actin cytoskeleton, helping cell spread by coupling matrix ligand to intracellular cytoskeleton [15]. TEM-8 seems to organize actin filaments into bundles [51], suggesting that potential actin reorganization drives cell shape changes and spreading after PA attachment. In addition, TEM8-1, but not TEM8-2 interacts with the actin network which modulates affinity for PA and promotes its heptamerization [25,51,52]. An inverse correlation exists between TEM8 binding to actin and the amount of PA bound to receptors [51]. Cortical actin cytoskeleton seems to be required for heptamerization of PA when bound to TEM8-1 that is organized in an actin dependent manner, but not when bound to CMG2. Upon toxin binding, actin-TEM8 interaction is released, but actin remains essential for heptamerization and endocytosis of heptameric PA [25]. However, future studies are required to better understand the role of the cytoskeleton in toxin uptake and intoxication.

## 4. Anthrax Toxins and Disruption of the Cytoskeleton Network

### 4.1. LT Effects

**Figure 2:**
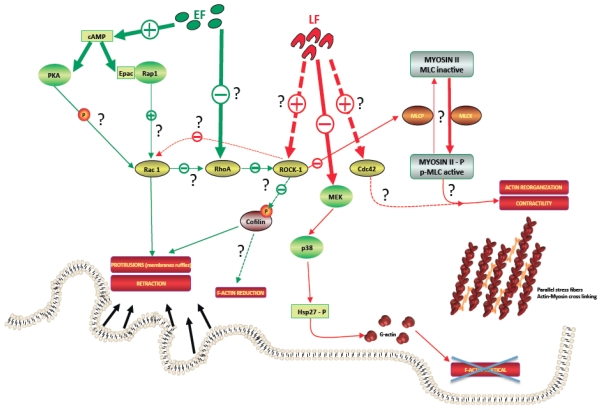
Effects of anthrax lethal and edema factors on cytoskeleton regulatory pathways.This hypothetical figure describes anthrax lethal factor (LF, red) and edema factor (EF, green) main targets along the regulatory pathways of cytoskeleton. On the one hand, LF probably activates Cdc42 and/or ROCK. ROCK activation could increase Myosin Light Chain (MLC) phosphorylation, leading to cell contractility. In parallel, LF-Mitogen-Activated Protein Kinase (MEK) cleavage blocks the Hsp27 phosphorylation cycle, impairing actin monomer transport to an area of new actin filament assembly. On the other hand, EF increases the cAMP level which signals via PKA and/or Epac/Rap1, potentially leading to the activation of Rac1 and inducing protrusions and retraction of the membrane. In parallel, EF could inhibit RhoA, leading to a reduction of F-actin via cofilin activation.

The effects of LT on the cytoskeleton have been relatively recent discoveries. Most of the studies have focused upon actin in endothelial and epithelial cells, as well as immune cells like macrophage or neutrophils (Figure 2).

#### 4.1.1. Actin Network

One major issue when considering the effects of LT on various cells is its diverse effects. On one hand, after exposure to LT, human endothelium and lung epithelial cells progressively show mechanical stiffness [11], blebbing, and elongation [53,54]. These morphological changes correlate with re-organization of the actin cytoskeleton characterized by thick actin cables or actin stress fibers [53,54,55,56] parallel to each other, with loss of cortical F-actin [53,54] but increased central F-actin content [11,54] or increased actin filament assembly [11]. For many authors, unconventional formation and stabilization of stress fibers occurred in the presence of LT over a course of 6–24 h, *i.e.* (i) in the absence of direct activation of RhoA/ROCK pathway or Rac1 by LT [11,53]; (ii) with equivalent phosphorylation of both cofilin [11,53] and myosin light chain (MLC) [53]; (iii) requires host gene transcription modifications by LT to participate in the thick actin cable formation [11,53]. For Cdc42 activity, results are more divergent: Lehmann *et al.* observe an increase in active GTP-bound Cdc42 in LT-treated epithelial cells [11] while Rolando *et al.* show equivalent activity of Cdc42 in LT-intoxicated human endothelial cells [53].

In contrast to the previous studies, a recent study of Warfel *et al*. on lung microvascular endothelial cells shows actin stress fibers accompanied by increased MLC phosphorylation and cleavage of ROCK-1, leading to the activation of ROCK-1 after 72 h of LT exposure. ROCK-inhibitors seem to block LT-induced stress fiber formation and phosphorylation of MLC, suggesting an involvement of ROCK pathways in maintaining LT-induced stress fibers [56]. Consequently, RhoA/ROCK pathway is most likely not directly modulated by LT but may control the stability of these actin cables.

On the other hand, after 2 h, LT reduces the ability of neutrophils to spread and actin filament assembly at the leading edge [13,57]. Actin-based motility of the intracellular pathogen *Listeria monocytogenes* in HeLa cells is also affected by LT [14]. In proteomics studies on mouse macrophage, the beta isoform of actin is upregulated [58], but gamma actin is decreased [59]. The study by Nour *et al*. also describes LT-treated murine macrophages lacking actin cytoskeleton after 90 min [60].

All these studies may also implicate one or all of the three major MAPK pathways altered in LT intoxication [61]. The effect of LT on neutrophil actin assembly is independent of MEK1 inhibition [14], while inhibition of the p38-MAPK pathway by LT blocks the Hsp27 phosphorylation cycle, impairing actin assembly and chemotaxis [13].

Taken together, these opposite results suggest that p38 MAPK signaling and other pathways like Cdc42 or actin regulators, such as MLC or ROCK, may mediate LT effects on actin dynamics. Yet, the mechanism by which LT leads to this reorganization of the actin cytoskeleton remains unclear.

#### 4.1.2. Microtubule Network

While LT induces stress fiber formation in lung epithelial cells, it stabilizes microtubules, with multiple, highly active protrusions without formation of a leading edge and MTOC polarization [11]. However, proteome and DNA array analysis of mouse macrophages treated with LT induce a notable decrease of cellular tubulin [59,62], while LF alone does not appear to directly cleave tubulin [62]. DNA array analysis also indicates that microtubule-associating proteins expression (*i.e.*, kinesin motor-protein) is altered in LT-treated macrophages [62], suggesting an altered stability of the microtubule network in macrophages.

### 4.2. ET Effects

ET induces significant morphological and cytoskeletal changes in different mammalian cells [63], including macrophages [10,64,65], primary human microvascular endothelial cells (HMVEC) [12] and neutrophils [57] (Figure 2). Some morphological differences have been observed between cells after ET treatment: (i) formation of filopodial protrusions in mammalian cells [63,64] *versus* reduction in macrophage [10]; (ii) rounded morphology in macrophages and mammalian cells [10,63,64] *versus* flattened morphology in human microvascular endothelial cells (HMVEC) [12]. In any case, every cell type had reduced spread morphology, a lowered F-actin content and actin redistribution to the cell margin in a time-dependent manner [10,57,63,64].

Some results support a role for the cAMP-dependent Protein Kinase A (PKA) pathway in the reorganization of actin network [10,57,63,64]. PKA is a serine-threonine kinase that phosphorylates many cytoskeletal proteins, including actin, microtubules and intermediate filaments. The role of Exchange Protein Activated by cAMP (Epac) is less clear, as one study on mammalian cells suggest a lack of Epac activation [63], while another suggests an activation of Epac and Ras-proximate-1 (Rap1) pathway alone [12], or associated with PKA signaling [10]. The disruption in the balance of Epac/PKA activity seems to be responsible for these cytoskeleton’s effects. As for LT effects, it clearly appears that ET-related cytoskeleton disruption and intracellular signaling are cell type-dependent. So far, no studies have explored the potential effect of ET on microtubules.

Because of its biochemical adenylate cyclase activity, ET may be compared to toxins with identical functions: ExoY of *Pseudomonas aeruginosa* [66] or adenylate cyclase toxin of *Bordetella pertussis* [67]. *B. pertussis*, the causative agent of whooping cough, produces toxins including an adenylate cyclase (CyaA) which also possesses hemolytic activity. It appears clearly that CyaA causes morphological changes [68,69,70], including membrane blebbing in erythrocytes [70]. Recent work by Kamanova *et al*. demonstrated that the adenylate cyclase activity of CyaA causes transient and selective inactivation of the GTPase RhoA in mouse macrophages and activation of cofilin, leading to massive actin rearrangements. These latter effects wane faster at high toxin concentration and are accompanied by a formation of membrane extensions referred as lamellipodia or membrane ruffling [71]. Consequently, cAMP signaling of CyaA toxin rapidly halts complement-mediated phagocytosis of macrophages [71] and human neutrophils [69,72,73]. 

Soluble adenylate cyclase ExoY of *P. aeruginosa*, injected by a type III secretion system in a host also generates a cAMP pool in the cytosol and mimicked using a forskolin activated soluble adenylyl cyclase I/II (sACI/II) by several authors ([74] and reviewed in [75]). Activation of sACI/II or directly ExoY generates a large cAMP increase that induces endothelial cell retraction [74,75], with an intact cortical F-actin network and a decrease of MLC phosphorylation [74] and probably a role in bleb-niche formation in epithelial cells [76].

## 5. From the Cytoskeleton up to Cellular and Organ Disruption

Anthrax toxins have many effects on the cytoskeleton. We will examine here the consequences of cytoskeleton disruption on some key cell functions and at the organ level. We focus our analysis here on two paradigms of cell function and organ disruption: cell phagocytosis and vascular integrity.

### 5.1. *B. anthracis* Toxins, Cytoskeleton and Phagocytosis Disruption

Phagocytosis is one of the first innate defense mechanisms involved for pathogen scavenging. Moreover, phagocytosis activates the adaptive immune system by antigen presentation. As *B. anthracis* escapes so efficiently from the innate and adaptive immune response, phagocytosis disruption by anthrax toxins does not come as a surprise.

*In vivo*, LT inhibits primary peritoneal macrophages fluorescent microspheres phagocytosis after LT-intraperitoneal injection [77], even though cultured nonhuman primate alveolar macrophages are still capable of spore phagocytosis after LT treatment [78]. Recently, Kau *et al*. demonstrate that LT suppresses the phagocytosis of J774 macrophage cells at low doses without influencing the MAPK pathways during early infection [79], implicating another pathway. Nevertheless, these effects are unclear and do not affirm that phagocytosis is inhibited by LT.

The effect of ET on human neutrophils [80] or human macrophages [10] shows a reduction, even an inhibition, of the phagocytic capacity of these cells. This is largely due to an inability to undergo actin remodeling, required for phagocytic activities. However, it clearly appears that aspects of ET-related actin cytoskeleton on phagocytic capacities of *B. anthracis* Ames spores depend on the cell type [10].

The cytoskeleton disruption by anthrax toxins and potential consequences on phagocytosis is likely important during later stages of infection and would benefit pathogen survival. The ability of these toxins to reduce or inhibit phagocyte functions represents a dangerous immuno-evasive mechanism where the extracellular vegetative cells can rapidly multiply and be eliminated with difficulty.

### 5.2. Anthrax Toxins and Vascular Disruption

Corruption of vascular integrity by ET has been known since its discovery in the mid 1960s, as it was named after its effects observed following subcutaneous injection [81]. These observations could be related to clinical records in oro-pharyngeal anthrax, an uncommon form of the disease characterized by impressive neck swelling [82]. Interestingly, subcutaneous *versus* intravascular injection of ET are not symmetrical, as they do not lead to the same outcome. On one hand a local reversible edema is only observed, while on the other hand the toxin is lethal [83]. This observation has one corollary: toxins act differentially when in the blood lumen or out of the vessel. When both anthrax toxins enter the bloodstream, they corrupt vascular homeostasis leading to a cardio-vascular collapse (review in [8,84]). From a pathogen’s standpoint both toxins facilitate pathogen spread, as they breach the vascular barrier between the blood and tissues leading to bacilli proliferation in multiple organs (review in [84]). These results suggest that ET may have been selected throughout evolution for its effects at the last stage of the disease when it is released into the bloodstream. Recent studies have confirmed that LT opens the blood-brain barrier favoring meningitis, one of the most frequent complications of systemic anthrax [85,86].

Surprisingly, anthrax toxins target the vascular endothelial system but are still poorly described mechanistically (let alone the cardiac effects). LT disrupts *in vitro* vascular endothelial cells by increasing actin stress fibers and disorganizing VE-cadherin at the adherents junctions (AJ) gasket [87] and protein zona occludens-1 at tight junctions (TJ) [85]. *In vivo* LT increases vascular permeability in zebrafish [88]. ET has also significant disrupting effects on cadherin in AJ [89]. Strikingly, mechanistic insights have been brought by a group using *Drosophila* as a model to deconstruct host-pathogen interactions [90,91]. They have very elegantly shown that ET and LT vascular effects are caused through a coordinate disruption of Rab11/sec15 exocyst [89]. The exocyst complex regulates docking of specific cargo vesicles to AJ. By deregulating AJ, both toxins corrupt cytoskeleton organization and vascular permeability. Another effect of toxin on vascular permeability is the formation of transendothelial macro-aperture (TEM) [92]. Very recently ET has been shown to induce TEMs, thus increasing peripheral vascular permeability [93]. More interestingly, the cytoskeleton senses the formation of TEM through a protein called Missing in Metastasis (MIM) that senses *de novo* membrane curvature, ultimately driving Arp2/3-dependent actin polymerization [93]. These very well designed studies clearly demonstrate the role of cytoskeleton in maintaining vascular integrity that is challenged by ET.

## 6. Conclusions-Perspectives

As we have shown here, the cytoskeleton emerges as the central target of anthrax toxin effects. As the cytoskeleton plays a crucial role in eukaryote cell biology, it is not surprising that toxins act on the central coordinator of cell life. New tools are emerging as the cytoskeleton is studied for its central role in cancer and development biology. Moreover, whereas ET and LT synergize their action against host innate immunity [94], the simultaneous effects of these two toxins on the cytoskeleton have only been examined by Szarowicz *et al*. [57]. This latter study suggests that the combination of ET and LT results in additive reduction in F-actin content, suggesting that these toxins impair actin assembly by different signaling pathways. So, it appears necessary to better understand the simultaneous effects of the two toxins on the cytoskeleton and the functional consequences for phagocytes. Thus, these elements would address the issue of whether ET and LT act in synergy or opposition to the cytoskeleton of phagocytes. A better understanding of *B. anthracis* toxin effects may allow the discovery of new inhibitors and better ways to treat anthrax. Outlined here are new avenues for researchers covering central questions that remain to be answered, such as the description of toxin effects as well as their potential synergistic effects upon the actin cytoskeleton.
